# Investigation of Antioxidant Properties of Propolis Products Collected from Different Regions

**DOI:** 10.3390/ijms27021046

**Published:** 2026-01-21

**Authors:** Aynur Cetin, Sena Bakir, Tugba Ozdal, Esra Capanoglu

**Affiliations:** 1 Department of Food Engineering, Faculty of Chemical and Metallurgical Engineering, Istanbul Technical University, 34469 Maslak, Istanbul, Türkiye; aynurcetin@itu.edu.tr; 2 Scientific Biosolutions (SBS-Bilimsel Bio Çözümler A.Ş. Bee’O Propolis), 34887 Sancaktepe, Istanbul, Türkiye; 3 Department of Gastronomy and Culinary Arts, Tourism Faculty, Recep Tayyip Erdogan University, 53400 Ardesen, Rize, Türkiye; sena.bakir@erdogan.edu.tr; 4 Department of Genetics and Bioengineering, Faculty of Engineering and Natural Sciences, Istanbul Okan University, 34959 Tuzla, Istanbul, Türkiye; tugba.ozdal@okan.edu.tr

**Keywords:** propolis, antioxidant, LC-MS analysis

## Abstract

Propolis, a sticky bee hive product collected from resinous plant sources by *Apis mellifera* bees, exhibits a wide range of biological and pharmacological properties, primarily attributed to its rich composition of bioactive constituents, including phenolic acids, esters, and flavonoids. In this study, the antioxidant properties of 76 liquid propolis solutions from 18 different countries were investigated based on their dry matter, total phenolic and total flavonoid contents, antioxidant capacities, and phenolic profiles. The antioxidant activities of propolis from various geographic regions, including Latvia, Croatia, New Zealand, San Marino, Russia, France, Romania, Italy, Estonia, Brazil, Belgium, Germany, Slovenia, Japan, the United States of America (USA), the United Arab Emirates (UAE), Spain, and Korea, were compared. Total phenolic and flavonoid contents, as well as total antioxidant capacity (Cupric Reducing Antioxidant Capacity—CUPRAC method), were analyzed by spectrophotometry, and the major constituents were investigated by liquid chromatography–mass spectrometry (LC-MS/MS). Antioxidant test results indicated that 29 products scored below 10 mg Trolox equivalent (TE)/mL, and only 14 were scored above 100 mg TE/mL. The results showed that the total phenolic content of the samples ranged from 0.1 to 107.5 mg Gallic acid equivalent (GAE)/mL, while total flavonoid content varied between 0.1 and 174.5 mg Catechin equivalent (CE)/mL. Based on the CUPRAC assay, total antioxidant capacity values ranged from 0.1 to 492.3 mg TE/mL. Among the 76 analyzed samples, nine products exhibited antioxidant capacity values exceeding 150 mg TE/mL. In all of these samples, phenolic profiling confirmed the presence of propolis, and the analytical results were consistent with the information declared on the product labels. Hence, this study provides a comprehensive, real-market evaluation of commercial propolis products by integrating spectrophotometric assays with LC-MS-based targeted metabolomics profiling, highlighting formulation- and product type-driven differences in phenolic composition and antioxidant capacity beyond geographical origin.

## 1. Introduction

Propolis is a natural resinous material collected by honeybees (*Apis mellifera*) from the buds of leaves, exudates, and cracks in the bark of various living plants [1]. The substance has a distinctive odor and a yellow-green to dark brown color depending on the source and age. Propolis is a word derived from the Greek pro = for or in defense and polis = the city, referring to the defense of the hive and revealing how bees use it in their hives. Bees use propolis to seal gaps in the honeycombs, smooth internal walls, and encapsulate the corpses of intruders that die inside the hive to prevent their decomposition [2]. Moreover, the resulting product, propolis, protects the hive against diseases caused by microbes and predators that coexist in the same environment [3], while also providing thermal isolation of the hive [1,4,5]. It is additionally used to clean the honeycombs before honey and its offspring are placed into the cells [3]. In this way, propolis helps maintain colony health by preventing putrefactive processes that may develop within the hive [4,5].

Propolis has long been recognized for its therapeutic properties, and it was extensively used in the traditional medicine of ancient Greek, Roman, and Egyptian civilizations [6]. Its applications as an antibacterial, antifungal, and antiviral agent have since expanded worldwide [7]. Owing to these diverse bioactive benefits, propolis is now incorporated into various food products and nutritional supplements, either in its raw form or as standardized extracts [8].

The resinous structure of propolis makes it hard and brittle at room temperature in its unprocessed form. Due to its unfavorable rheological properties, propolis cannot be consumed raw, and its direct dispersion in water is highly challenging. Most of the bioactive components of propolis are readily soluble in ethanol but poorly soluble in water; therefore, propolis is commonly commercialized as a hydroalcoholic extract [9], including ethanol, olive oil, glycerol, polyethylene glycol, dimethylsulfoxide, and mineral salts, depending on the intended application [10,11]. Nevertheless, the presence of alcohol limits its use for health, religious, or age-related reasons; it may also cause adverse effects when ingested or applied as a spray, including allergic reactions, irritability, agitation, headaches, nausea, and dizziness. To minimize such side effects, propolis is also marketed in powder form, as chewing gum, mouth spray, syrup, and capsules [12]. Extracts obtained with different solvents are incorporated into toothpaste, dental floss, mouthwash formulations, and numerous pharmaceutical and cosmetic products such as creams, roll-ons, serums, ointments, lotions, and solutions [11]. Additionally, propolis is available in tablet, powder, pastille, chewing gum, and various capsule forms. In complementary medicine, propolis extracts are used as sprays, powders, and drops to treat a variety of illnesses, including asthma, colds and the flu, and gastrointestinal disorders [13,14]. In order to produce an effective extract, obtaining a product with high biological activity is essential. An equally important factor in preparing propolis extract is selecting a solvent that is non-toxic, suitable for consumption, removable after extraction when necessary, and compatible with safe daily intake levels. Considerations also include its absorption in the body, the metabolites formed, and their subsequent excretion [3].

Propolis contains a variety of chemical constituents, such as flavonoids, phenolic acids and their esters, aromatic aldehydes and alcohols, terpenoids, steroids, and amino acids [15]. It depends on the geographical and botanical origins of the propolis, as well as the species of bees involved regarding its composition [16]. Due to geographic differences, propolis samples collected from Europe, South America, and Asia exhibit different chemical compositions. Many flavonoids and phenolic acid esters are present in propolis from Europe and China. The differences in propolis composition make it difficult to assess its quality, as available chemical methods are insufficient for propolis quality control [17,18].

Recent studies increasingly emphasize that antioxidant capacity measurements should be interpreted together with compound-level compositional data obtained through LC–MS-based profiling and chemometric approaches, particularly for complex natural products such as propolis [19,20,21]. Integrative strategies combining spectrophotometric antioxidant assays with targeted LC–MS/MS analysis have been shown to improve the interpretation of functional readouts by linking antioxidant performance to specific phenolic constituents and formulation-dependent variability [20,22]. This approach is especially relevant for commercially available propolis products, where differences in extraction solvents, product forms, and processing conditions can substantially influence both measured antioxidant capacity and underlying chemical composition [23]. Accordingly, combining CUPRAC-based antioxidant assessment with targeted LC–MS profiling represents an emerging analytical direction for evaluating the functional and compositional heterogeneity of commercial propolis products [19,20,23]. While antioxidant capacity measurement provides an integrated measure of antioxidant redox capacity, targeted LC–MS analysis enables compound-level resolution of the phenolic constituents underlying this activity, allowing antioxidant performance to be interpreted within a formulation-dependent framework.

**Figure 1:**
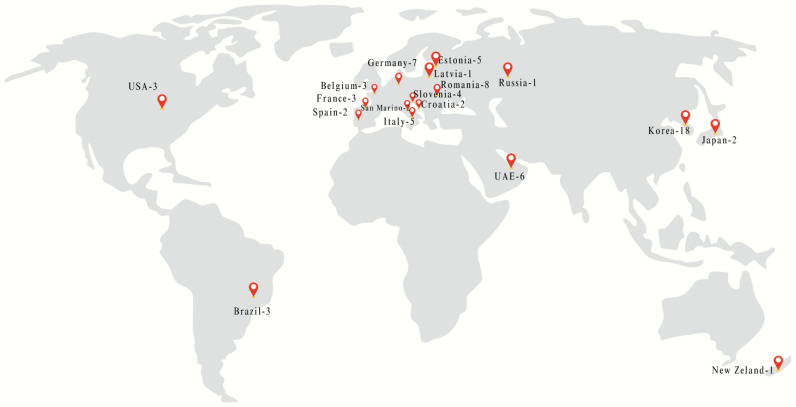
The distribution of propolis regions and collection amounts.

Most studies in the literature focus on the characterization of propolis, its biological activities, and the influence of its chemical composition. Various extraction techniques—ranging from conventional methods to more ecologically friendly approaches—have been reviewed [16], along with analytical methods for identifying its bioactive constituents and the chemogeographic variations in its composition [24]. In one study, seven propolis samples from different regions of Bulgaria and eight commercial propolis extract products from various European countries were examined [25]. High-performance liquid chromatography coupled with photodiode array detection was used to determine the phenolic profiles of propolis samples from Poland, Uruguay, Türkiye, and Romania, and the antioxidant activity of the extracts was evaluated using in vitro DPPH and ABTS assays [26]. However, no previous studies have comprehensively examined different forms of propolis products. Despite extensive research on the chemical composition and biological activities of raw propolis and standardized extracts, comparatively little attention has been paid to commercially available propolis products formulated in different liquid forms. Most existing studies primarily emphasize botanical or geographical origin, while the potential influence of formulation type, solvent composition, and product processing on molecular phenolic profiles remains insufficiently explored. Commercial propolis products are marketed in diverse formulations, such as drops, sprays, and syrups, each of which uses distinct extraction solvents and matrix conditions that may influence phenolic composition, stability, and antioxidant behavior. However, molecular-level evidence addressing how these formulation differences shape metabolite profiles under real-market conditions is limited. Addressing this gap is essential to understanding variability across commercial products and to improving quality assessment and consumer transparency. To the best of our knowledge, this study is the first to investigate the in vitro antioxidant properties of propolis collected from various regions across multiple countries and to analyze the individual chemical constituents in different propolis product forms (Figure 1). Three analytical systems were used to evaluate propolis samples from diverse geographic origins: total phenolic content (TPC) using the Folin-Ciocalteau method, total flavonoid content (TFC) using the aluminum colorimetric method, and antioxidant capacity using the CUPRAC method. In addition, several compounds in the propolis extracts were identified and quantitatively determined using ultra-performance liquid chromatography-tandem mass spectrometry (UPLC-MS/MS).

## 2. Results

### 2.1. Dry Matter and pH Values of Collected Samples

The moisture content of a propolis sample is influenced by storage and handling conditions and should therefore be considered in all evaluations. All samples were analyzed for pH and dry matter, and the results are summarized in Table A1. Due to variations in formulation and extraction procedures, the pH values of the products ranged from extremely acidic to slightly alkaline. Compared with syrup formulations, drop and spray products exhibited a broader pH range, indicating greater variability in their chemical composition. In contrast, syrup samples generally showed a narrower pH range, suggesting a more stable and uniform formulation.

Samples also differed significantly in their dry matter content. Drop products exhibited lower and more variable dry matter content, whereas syrup products consistently showed higher dry matter values. Due to variations in solvent systems and formulation techniques, spray products had intermediate dry matter levels with significant variability. In comparison to samples extracted using alcohol- or glycol-based solvents, those extracted with aqueous-based solvent systems (such as water, water–glycerin, or water–ethanol combinations) typically showed higher dry matter content. Highly varied pH and dry matter values were found in products with undefined solvent information, suggesting possible heterogeneity in formulation techniques.

The observed diversity in metabolite profiles and antioxidant activities reported in the following sections can be better understood in light of these physicochemical differences.

### 2.2. Spectrophotometric Analysis

A total of 76 propolis samples from 18 countries were analyzed for TPC, TFC, and antioxidant capacity (Table 1). Significant heterogeneity was observed among samples, with large ranges in TPC, TFC, and CUPRAC values across regions. Extensive ranges were observed in certain regions, characterized by both extremely high and extremely low values, indicating substantial heterogeneity among commercial products. Conversely, nations with smaller ranges typically had lower maximum values, suggesting that their products were less phenolic-enriched but more consistent. The significance of phenolic composition, especially flavonoid concentration, in determining antioxidant capacity is highlighted by the fact that high CUPRAC values were not always linked to the highest TPC values.

**Figure 2:**
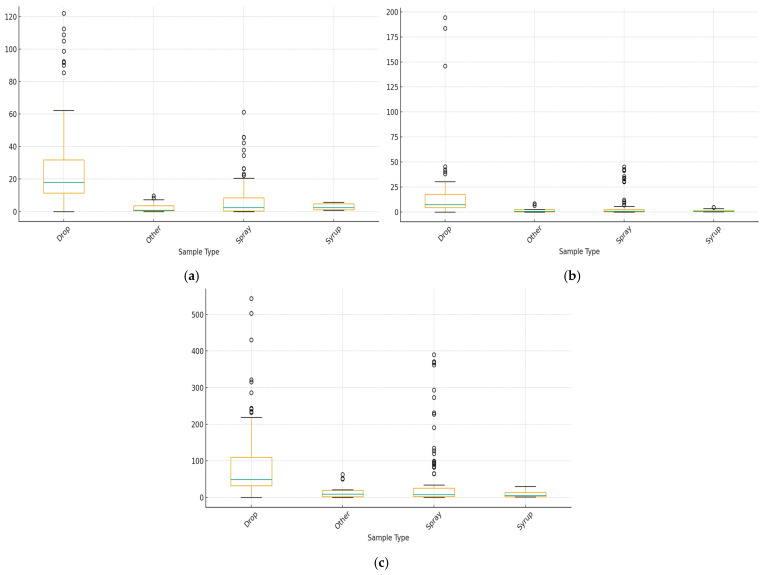
Comparison of spectrophotometric data results of samples. (**a**) TPC, (**b**) TFC, and (**c**) CUPRAC of samples. Results were given as mg gallic acid, mg catechin, and mg Trolox equivalent per mL of sample, respectively (*p* < 0.05).

The TPC varied significantly between the various sample types (Figure 2a). Drop samples had the highest median TPC values, as indicated by the boxplot, and multiple high TPC values indicated significant phenolic enrichment. Syrup and other treatments consistently exhibited reduced TPC values, whereas spray samples showed intermediate amounts. Tukey’s post hoc comparisons showed that drop samples were significantly higher than spray, syrup, and other (*p* < 0.05), and a one-way ANOVA indicated a significant effect of sample type on TPC values (*p* < 0.001). This pattern suggests that while syrup and other samples continue to have relatively low TPCs, droplet application significantly increases the accumulation of phenolic chemicals.

Additionally, there were notable differences in TFC across sample type groups (Figure 2b). The highest median TFC values were observed in drop samples, indicating higher concentrations of flavonoid compounds. While others and syrup tended to cluster at the lower end of the distribution, spray samples showed moderate values with wide variability. Tukey’s analysis showed that drop samples had considerably higher TFC values than all other treatment groups (*p* < 0.05), and ANOVA results indicated a statistically significant effect of sample type on TFC (*p* < 0.001). While syrup and other samples have relatively lower flavonoid levels, our results support the theory that droplet application increases flavonoid retention or extraction efficiency.

The overall antioxidant capacity, as indicated by CUPRAC values, showed a similar pattern to that of TPC and TFC (Figure 2c). The CUPRAC values of drop samples were significantly higher, with several outliers and high median scores indicating robust antioxidant capacity. While the antioxidant capacity of spray samples was moderate, that of syrup and other treatments was consistently low. ANOVA showed that sample type had a highly significant effect on CUPRAC (*p* < 0.001), and post hoc comparisons indicated that spray samples differed significantly from the lowest categories (*p* < 0.05). Drop samples were considerably higher than syrup and others. This significant difference suggests that the type of sample treatment, especially droplet application, is strongly associated with to antioxidant capacity.

**Figure A1:**
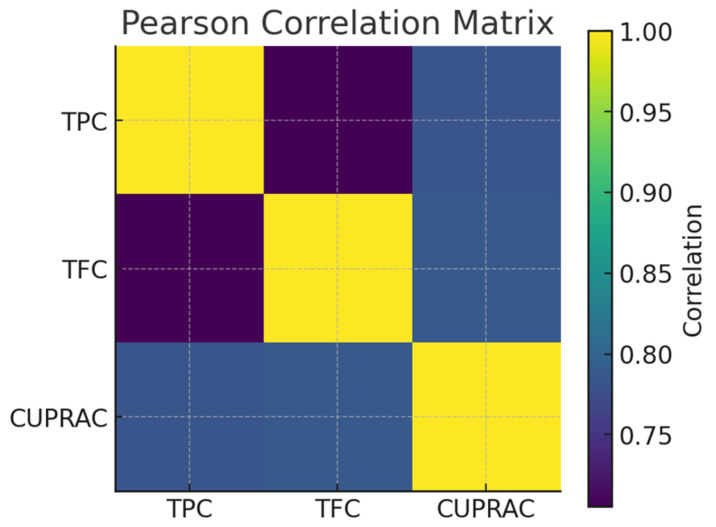
Pearson correlation matrix of samples based on the spectrophotometric data results.

Strong positive correlations between TPC, TFC, and CUPRAC values were found using Pearson correlation analysis (*r* = 0.70–0.79), as shown in Figure A1. TFC and CUPRAC showed the highest correlation (*r* = 0.787), suggesting that flavonoids significantly contribute to total antioxidant capacity. In a similar vein, there was a strong correlation between TPC and CUPRAC (*r* = 0.784), indicating that phenolic components are the main factors influencing the samples’ capacity to reduce radicals. In line with other findings in fruit and plant matrices, these strong intercorrelations suggest that propolis samples with higher phenolic and flavonoid concentrations exhibit stronger antioxidant properties.

**Figure 3:**
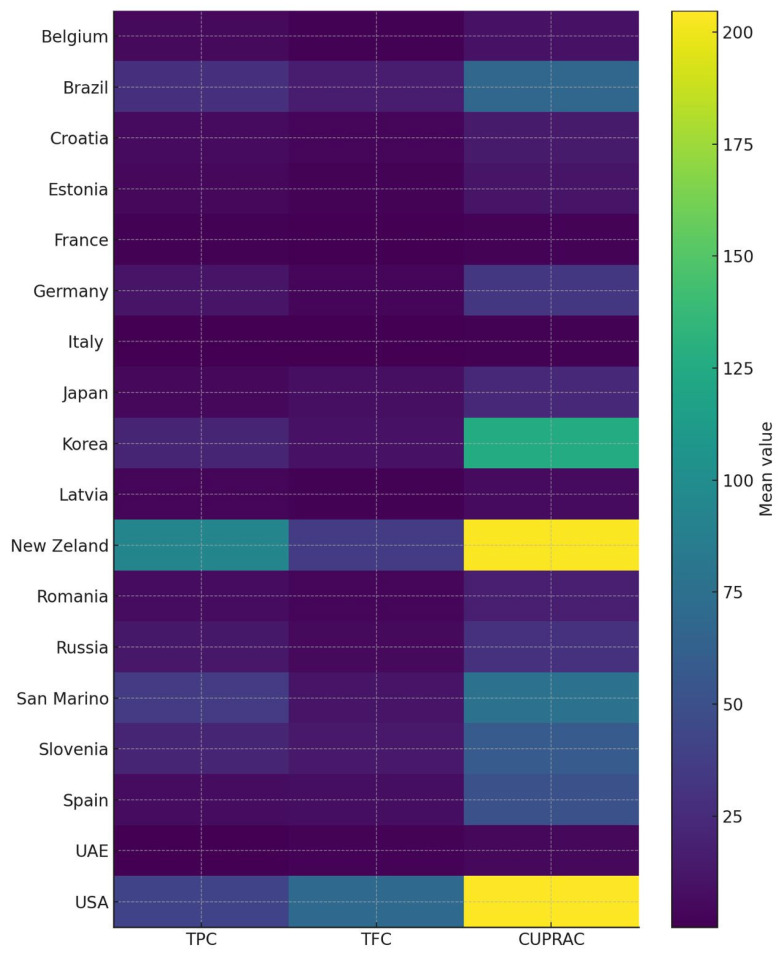
Heatmap demonstration of the spectrophotometric data results of samples according to the comparison of the country of origin.

All studied antioxidant parameters showed significant regional variation (Figure 3). Italy, France, and Latvia consistently had the lowest mean values of TPC, TFC, and CUPRAC, whereas New Zealand, the USA, Brazil, and Korea had the highest. Country had a substantial impact on all three variables, as indicated by a one-way ANOVA (*p* < 0.001). Numerous pairwise differences were found using Tukey’s post hoc comparisons, and samples from New Zealand were significantly higher than those from most other nations (*p* < 0.05). Compared with samples from European and Middle Eastern nations, those from the USA and New Zealand show notably higher phenolic content and antioxidant capacity. These findings show that phenolic content and antioxidant activity are significantly influenced by the nation of origin, likely reflecting variations in sample processing, climate, and variety.

### 2.3. LC-MS Data Results

**Figure 4:**
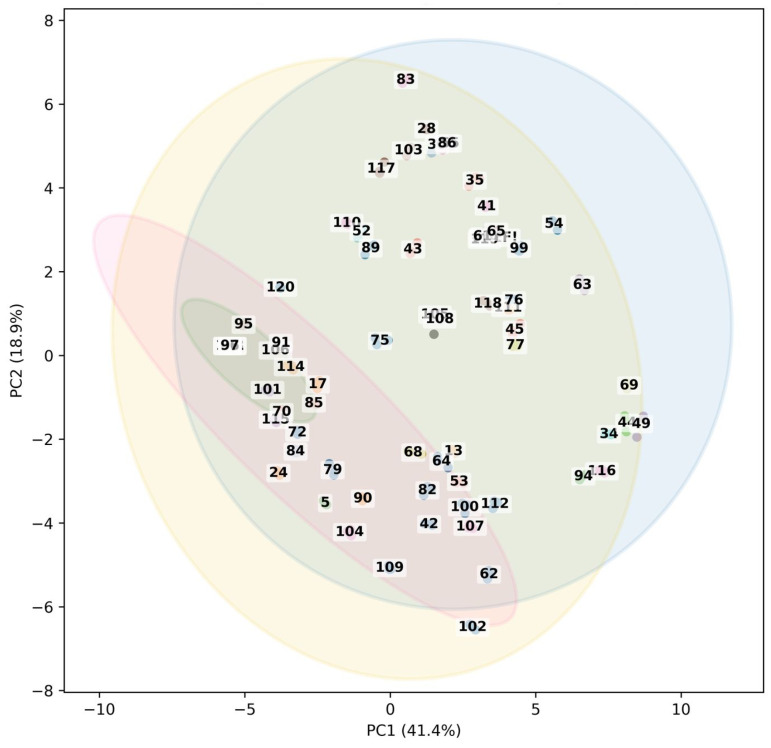
PCA score plot based on LC–MS metabolite profiles. Each point represents an individual commercial propolis product and is labeled with its corresponding sample identifier, as listed in Table A1. The sample identifiers are non-consecutive because they reflect original commercial product codes. Samples are colored according to country of origin, while confidence ellipses (95%) illustrate the distribution of product types, including drop (blue), spray (yellow), syrup (pink), and other (green). The plot highlights overall compositional trends rather than compound-level information.

The overall variation among samples based on their phenolic-targeted metabolite profiles was visualized using Principal Component Analysis (PCA) (Figure 4). The two-dimensional score plot accounted for over half of the dataset’s variability, with the first two principal components explaining 41.4% (PC1) and 18.9% (PC2), respectively, and the first two principal components accounted for 60.3% of the variance, suggesting that a significant amount of metabolomic variability could be explained in a two-dimensional space.

Replicate samples from the same group that cluster together show strong analytical reproducibility and dependable LC-MS data quality. The observed separation likely reflects treatment-dependent alterations in antioxidant and phenolic signatures, as the investigated chemicals include important phenolic acids and flavonoids (e.g., caffeic, ferulic, gallic, and p-coumaric acids, apigenin, and naringenin). Clear separation between treatment groups was observed by color-coding the samples by product type. Drop samples, in particular, clustered closely, indicating a consistent metabolic response to this treatment. The distribution of the spray and syrup samples, on the other hand, was wider along the PC1 axis, suggesting greater metabolic variability and a more pronounced effect of these treatment techniques on phenolic content. The fact that the other samples were located in different peripheral areas of the PCA plot suggests that this group might reflect a distinct metabolic phenotype with biochemical traits that differ significantly from those of the other treatment groups.

Group—Country information was added in sample labels, enabling the depiction of both treatment effects and geographic origin. The separation patterns were mainly driven by product type rather than by country of origin; however, some country-specific clustering was observed. This implies that metabolite composition is more strongly influenced by sample treatment than by regional considerations. PCA loadings showed that flavonoids and phenolic acids, such as apigenin, galangin, pinocembrin, quercetin, chrysin, naringenin, as well as p-coumaric, caffeic, and ferulic acids, were the main drivers of PC1. These chemicals’ significant positive loadings imply that PC1 captures variation in total phenolic and flavonoid abundance across samples. Therefore, samples with positive PC1 scores can be considered comparatively enriched in these bioactive chemicals, while samples with negative PC1 scores show lower relative quantities. PC2, on the other hand, captured secondary variation associated with compositional changes rather than overall metabolite abundance. PC2 indicates changes in the relative balance between phenolic acids and specific flavonoid-related compounds, with high positive loadings for ferulic, p-coumaric, and caffeic acids, and negative contributions from chrysin and CAPE. This pattern implies that, rather than variations in total concentration, variation along PC2 is driven by changes in metabolite composition and the proportional contributions of compound classes across samples. Importantly, the loading patterns observed along PC1 and PC2 also provide functional insight into antioxidant behavior. PC1, driven by flavonoids and phenolic acids with high redox activity, reflects variation in overall reducing capacity. In contrast, PC2 captures differences in the relative contribution of specific phenolic acids and flavonoids with distinct redox potentials, which may influence antioxidant performance independently of total phenols. This interpretation supports the observation that antioxidant capacity is governed not only by phenolic quantity but also by compound-specific redox characteristics and compositional balance.

Multivariate metabolite profiles and TPC, TFC, and antioxidant capacity (CUPRAC) showed a consistent pattern when combined with PCA results. Higher TPC and TFC values typically correlated with samples on the positive side of PC1, which was driven by significant loadings of flavonoids and phenolic acids, suggesting an enrichment of phenolic chemicals in these samples. Since these compound classes make up a considerable portion of the total phenolic and flavonoid contents, the strong contributions of flavonoids (such as apigenin, galangin, pinocembrin, quercetin, and chrysin) and phenolic acids (such as p-coumaric, caffeic, and ferulic acids) to PC1 support the trends in TPC and TFC measurements. The substantial contributions of flavonoids (apigenin, galangin, pinocembrin, quercetin, and chrysin) and phenolic acids (p-coumaric, caffeic, and ferulic acids) to PC1 support the trends in TPC and TFC measurements because these compound classes account for a large portion of the total phenolic and flavonoid contents. Opposite to PC1, variation along PC2 was linked to changes in phenolic component composition rather than total abundance. Variability in CUPRAC responses did not always correlate with changes in TPC or TFC values along this axis, indicating that the relative balance between phenolic acids and certain flavonoids may affect antioxidant activity.

## 3. Discussion

In this study, 76 propolis extracts were investigated for TPC, TFC, and antioxidant capacity in conjunction with LC-MS-based targeted metabolomic profiling. This approach offers supplementary insights into the variables influencing the bioactive components of the examined materials. An analysis of the physicochemical data shows that differences in pH values between samples are related to the composition of the extraction solvent. While samples using alcohol- or glycol-based solvents displayed a wider pH range, products manufactured with aqueous-based solvents frequently showed pH values within a narrower acidic-to-near-neutral range. These findings imply that the chemical environment of the products may be influenced by the composition of the extraction solvent, thereby affecting metabolite stability and extraction effectiveness. Physicochemical parameters such as pH play a critical role in both the chemical behavior of polyphenols and the interpretation of antioxidant measurements. Changes in pH influence the ionization state of hydroxyl groups in phenolic compounds, with higher pH promoting deprotonation and thereby enhancing electron-donating capacity [27]. At the same time, several studies have shown that polyphenols can remain chemically stable under mildly acidic conditions; for example, tea epicatechins exhibit high thermal stability at pH 5, with only limited degradation after prolonged heating [28]. The performance of spectrophotometric antioxidant assays is also strongly influenced by pH conditions. In this context, the CUPRAC assay operates at near-physiological pH, offering favorable redox potential, reagent stability, and accessibility for both hydrophilic and lipophilic antioxidants [29]. Proton dissociation of phenolics under more basic conditions may increase reducing capacity, whereas protonation under acidic conditions may reduce electron transfer efficiency [30]. Extraction solvent composition further contributes to variability in phenolic profiles and antioxidant behavior. While several studies have reported higher phenolic extraction efficiency and antioxidant activity for ethanol–water mixtures compared to aqueous solvents [31], others have demonstrated that water extracts of propolis with high phenolic content can also exhibit strong reducing power and radical-scavenging activity [32]. These contrasting findings indicate that solvent effects on antioxidant properties depend primarily on phenolic composition rather than on solvent type alone. In addition, moisture content may influence antioxidant measurements by affecting extract concentration and matrix effects. Taken together, these considerations support the inclusion of pH and moisture content in the present study as contextual parameters that aid interpretation of antioxidant data, rather than as direct indicators of antioxidant activity.

In TPC, TFC, and CUPRAC, drop samples showed the most significant variability and median values, whereas syrup and other samples consistently displayed lower amounts. A moderate improvement in antioxidant properties was indicated by the spray samples’ intermediate behavior, which included several high-value outliers. The ANOVA and Tukey post hoc tests support these patterns, indicating that the sample treatment has a substantial impact on phenolic content and antioxidant capacity.

In the meantime, the TPC, determined by the Folin–Ciocalteu method with gallic acid as the standard, also indicates a high concentration of phenolic compounds in the sample under study. According to another study, the phenolic content of the ethanolic extract of *Tetragonula* sp. propolis was 150 µg GAE/mL [33]. According to Molnár et al. [34], the TPC of propolis from various parts of Europe (Hungary and Bulgaria) ranged roughly between 104 and 287 GAE/g; meanwhile, DPPH radical scavenging activity ranged between 102 and 287 mg ascorbic acid equivalents/g. TFC ranged from 16.90 to 46.60 g quercetin equivalent/100 g dried sample weight in studies of a 70% ethanolic extract of stingless bee propolis samples from several Indonesian locations [35]. According to another study, the TFC of Indian stingless bee propolis was 48.00 µg quercetin equivalent/mL [36]. According to Kurek-Górecka et al. [37], propolis contains a variety of phenolic chemicals, primarily flavonoids. The diverse regional floras that honeybees choose to collect are the leading cause of the variances in flavonoid compounds found in propolis. The phenolic chemicals in propolis samples may vary depending on the time of collection, seasonal variations, and climate change. Four propolis samples were gathered from various parts of China: Yingchun, Heilongjiang Province (YHP); Changge, Henan Province (CHP); Raohe, Heilongjiang Province (RHP); and Linqing, Shandong Province (LSP). CHP > RHP > LSP > YHP is the DPPH free radical scavenging activity. Increased phenolic content is associated with increased DPPH activity [38]. Because of their biological role in scavenging reactive oxygen species, phenols in propolis’ ethanolic extracts contribute to its antioxidant activity [39]. It should be emphasized that spectrophotometric antioxidant assays reflect chemical reducing capacity under defined in vitro conditions and do not directly predict biological or in vivo antioxidant effects. Therefore, the antioxidant data presented in this study are interpreted as indicators of chemical antioxidant potential rather than biological efficacy. The observed differences among samples provide insight into variability in redox-active phenolic composition, underscoring challenges related to biochemical consistency and standardization among commercial propolis products.

An effective method for analyzing natural compounds is LC-MS. The MS analytical approach’s high sensitivity offers the possibility of finding novel minor constituents that are challenging to obtain through traditional methods. The phenolic constituents of northeastern Portuguese propolis were investigated in a prior study using a combination of offline HPLC and electrospray ionization (ESI) MS in the negative ion mode [40]. This method enabled the characterization of 37 phenolic compounds, encompassing typical phenolic acids and flavonoids found in temperate zone propolis, along with novel methylated, esterified, and/or hydroxylated derivatives of common poplar flavonoids and pinocembrin/pinobanksin derivatives featuring a phenylpropanoic acid derivative moiety in their structure [40]. 40 propolis samples from Portugal, a nation renowned for its botanical diversity, were analyzed, revealing 76 polyphenols. The predominant type was common temperate propolis, characterized by typical phenolic compounds such as flavonoids and their methylated/esterified derivatives, as well as phenylpropanoid acids and their esters. Additionally, an atypical propolis variant was identified, distinguished by an unusual composition of quercetin and kaempferol glycosides, some of which have not been previously documented in propolis. This analysis employed liquid chromatography with diode-array detection coupled with electrospray ionization tandem mass spectrometry [41]. Table A2 presents the quantitative distribution of the targeted metabolite composition in selected samples. Targeted metabolites—phenolic acids, including p-coumaric acid, gallic acid, caffeic acid, CAPE, ferulic acids; flavones, including chrysin and apigenin; flavanones, including pinocembrin and naringenin; flavanols, including galangin, catechin, and quercetin—were identified, and quantities of collected propolis samples were compared with each other in the scope of this study. The targeted analytes, such as chrysin, pinocembrin, galangin, caffeic acid, ferulic acid, apigenin, naringenin, and CAPE, are commonly identified in propolis phenolic profiles and have been associated with antioxidant and bioactive potential in the literature. For example, chrysin, pinocembrin and galangin have been reported as major flavonoids in European and other propolis types, contributing to phenolic content and antioxidant activity [42], while propolis phenolic compounds including caffeic acid and CAPE are widely recognized for their contribution to bioactivity [43]. Overall, propolis extracts rich in these compounds typically exhibit strong antioxidant capacity across diverse geographic origins [37]. The targeted LC–MS/MS approach was designed to quantify a selected panel of phenolic acids and flavonoids that are frequently reported as major bioactive constituents of propolis and commonly associated with antioxidant capacity. Compound selection was based on their reported prevalence in propolis matrices and their relevance to redox-active phenolic composition rather than comprehensive metabolome coverage. Beyond their antioxidant properties, flavonoids, including chrysin, pinocembrin, and galangin, have been shown to modulate biological pathways at the level of signaling, such as inflammatory, apoptotic, and oxidative stress-responsive pathways [44]. According to recent thorough assessments, formulation-dependent parameters that affect stability, bioavailability, and cellular absorption significantly influence these compounds’ biological relevance [45]. When evaluating functional results, it is important to consider the compound-specific diversity in commercial propolis formulations, which may extend beyond antioxidant screening. When paired with LC-MS data, multivariate metabolite profiles as well as TPC, TFC, and antioxidant capacity (CUPRAC) displayed a consistent pattern. Flavonoids and phenolic acids in samples were generally connected with higher TPC and TFC values, indicating an enrichment of phenolic compounds in these samples. These observations collectively support the consistency between LC–MS-based metabolite profiles and spectrophotometric measurements. Changes in TPC or TFC values did not always correlate with variations in CUPRAC responses in PCA, suggesting that the relative balance between phenolic acids and specific flavonoids may influence antioxidant activity. Importantly, LC–MS data indicate that antioxidant capacity is not driven solely by total flavonoid abundance, but rather by the relative contribution of specific phenolic acids and flavonoid subclasses with distinct redox properties. Phenolic acids such as caffeic and ferulic acid, together with certain flavonoids, are known to differ in electron-donating capacity and redox potential [46], which may explain why variations in CUPRAC responses do not always parallel changes in TPC or TFC. This compound-level behavior highlights that antioxidant performance reflects qualitative compositional balance rather than quantitative phenolic enrichment alone. Recent focused reviews further emphasize that such compound-specific behavior is strongly influenced by molecular structure and formulation context, which together modulate redox activity, stability, and biological relevance [47]. In this framework, the compound-level trends observed in the present study align with contemporary perspectives on flavonoid functionality beyond bulk phenolic content.

Geographical location, climate, and floral sources all have a substantial impact on propolis’s chemical composition, which in turn affects its biological diversity [48]. Furthermore, certain flowers in the immediate area of the beehives can contribute to the phytochemicals and bioactive compounds of the stingless bee propolis, indicating that the native vegetation surrounding the beehives influences the composition and chemical content of propolis [49]. Any pharmacological investigation of propolis without chemical characterization is irreproducible and meaningless, as its chemical composition varies widely across plants and geographic origins [50]. In the present study, although the country of origin showed statistically significant differences in spectrophotometric parameters, multivariate analysis indicated that these differences were less influential than sample type-related effects. This finding suggests that, under real-market conditions, formulation characteristics may outweigh geographical origin in shaping the phenolic composition and antioxidant behavior of commercial propolis products. This observation is consistent with previous reports highlighting the chemical complexity of propolis; however, the present findings emphasize the dominant role of formulation-related factors over geographical origin. It should be noted that the current study’s country-based comparisons are highly susceptible to regional variations in sample sizes, which can affect statistical confidence and limit the applicability of regional trends. Accordingly, observed country-related differences should be interpreted as indicative rather than definitive, reflecting exploratory patterns within commercially available products rather than intrinsic country-specific compositional profiles.

It should be acknowledged that the analyzed samples represent commercially available propolis products that differ substantially in formulation, solvent systems, processing, and labeling practices within the scope of this study. The observed differences should therefore be interpreted as reflecting formulation-driven and market-level heterogeneity in chemical profiles, rather than as evidence of intrinsic compositional superiority or biological efficacy. However, the importance of this study also comes from this, and provides insightful data on ready-to-use commercial samples’ content to consumers who have concerns about this issue. It should be noted that this study enables LC–MS/MS analysis targeted at a defined subset of phenolic compounds and does not represent a comprehensive untargeted metabolomic profiling of propolis. Therefore, conclusions regarding chemical composition are restricted to the selected phenolic markers and should not be extrapolated to the full propolis metabolome. Untargeted approaches may provide complementary insights in future studies.

## 4. Materials and Methods

### 4.1. Propolis Samples

In this study, 76 propolis samples collected between 2016 and 2019 in the form of extracts (drop-29, spray-35, syrup-6 and other liquids-6), sold in stores, pharmacies and online shopping websites from 18 different countries. 18 samples from Korea (Seoul, Republic of Korea), 8 samples from Romania (Bucharest, Romania), 7 samples from Germany (Berlin, Germany), 6 samples from United Arabian Emirates (UAE) (Abu Dhabi, UAE), 5 samples each from Estonia (Tallinn, Estonia) and Italy (Rome, Italy), 4 from Slovenia (Ljubljana, Slovenia), 3 samples each from Brazil (Brasília, Brazil), USA (New York City, NY, USA), Belgium (Brussels, Belgium) and France (Paris, France), 2 samples each from Japan (Tokyo, Japan), San Marino (City of San Marino, San Marino), Croatia (Zagreb, Croatia) and Spain (Madrid, Spain), and, finally, 1 sample each from Russia (Moscow, Russia), Latvia (Riga, Latvia), and New Zealand (Wellington, New Zealand) were collected. All 76 commercial propolis samples analyzed in the study are reported in full in Table A1, which provides detailed information on sample origin, extraction solvent, product type, pH, and dry matter content. Sample numbers correspond to original commercial product identifiers and are therefore non-consecutive. All samples were stored under dry conditions at 4 °C until analyzed.

### 4.2. Chemicals

All chemicals and reagents used in the current study were analytical or HPLC grade. For the determination of total phenolic and flavonoid contents and antioxidant capacities, gallic acid (≥98%), catechin, ethanol (≥99.8%), acetonitrile (99.8%), Folin–Ciocalteu phenol reagent and neocuproine (Nc) from Sigma-Aldrich Chemie GmbH (Steinheim, Germany); methanol (≥99.9%), formic acid (≥98%), sodium carbonate (Na_2_CO_3_), sodium nitrite (NaNO_2_), sodium hydroxide (NaOH), copper (II) chloride (CuCl_2_) and ammonium acetate (NH_4_Ac) from Merck KGaA (Darmstadt, Germany); 6-hydroxy-2,5,7,8-tetramethylchroman-2-carboxylic acid (Trolox) and aluminum chloride (AlCl_3_) from Fluka Chemie (Buchs, Switzerland) were purchased.

All chemicals used as standards in LC–MS/MS analysis, including apigenin, cafeic acid, (-)-catechin, p-coumaric acid, naringenin, quercetin, ferulic acid, gallic acid, chrysin, pinocembrin, galangin and CAPE were obtained from Sigma-Aldrich Chemie GmbH (Steinheim, Germany).

### 4.3. Dry Matter and pH Value Determination

Propolis samples were weighed using an analytical balance with a precision of three digits (Precisa Model XM 50, Precisa, Dietikon, Switzerland). Similarly, pH values were measured with a VWR pH1000L pH meter (Wien Austria). Measurements were performed in triplicate for each sample, and the results were reported as percentage (%) dry matter.

### 4.4. Determination of Total Phenolic Content

TPC assay was conducted using Folin-Ciocalteau reagent according to the method modified from Turkmen et al. [51] using gallic acid as a standard. A mixture of 100 μL of the sample, 900 μL of distilled water, and 1.5 mL of 0.2 N Folin–Ciocalteu reagent was prepared and incubated for 5 min. Then 1.2 mL of 7.5% Na_2_CO_3_ solution was added to the reaction mixture. The absorbance was measured at 765 nm after incubation for 90 min at room temperature. TPC of samples was expressed as mg of GAE per mL of sample.

### 4.5. Determination of Total Flavonoid Content

TFC in samples was determined colorimetrically as described by Dewanto et al. [52] with minor modifications. A mixture of 1 mL of the sample and 300 μL of 5% NaNO_2_ was prepared and incubated for 5 min. Then 300 μL of 10% AlCl_3_ was added and mixed thoroughly. After 6 min, 2 mL of 1 M NaOH and 2.4 mL of distilled water were added. Absorbance of the mixture was measured at 510 nm. The TFC of samples was calculated as catechin equivalents from the calibration curve, and the results were expressed as mg of CE per mL of sample.

### 4.6. Determination of Total Antioxidant Capacity

The cupric reducing antioxidant capacity (CUPRAC) assay, as described by Apak et al. [29], was used to assess the total antioxidant activity of the samples. In the CUPRAC assay, the antioxidant solution, copper (II) chloride solution, neocuproine alcoholic solution, and an ammonium acetate aqueous buffer solution were mixed at pH 7.0. The CUPRAC assay is based on the reduction in Cu(II) to Cu(I), followed by complex formation with neocuproine, resulting in an absorbance maximum at 450 nm. For the CUPRAC assay, 1 mL each of CuCl_2_ solution (1.0 × 10^−2^ M), neocuproine alcoholic solution (7.5 × 10^−3^ M), NH_4_Ac buffer solution at pH 7.0, and finally 1 mL water was added to 100 µL of the sample. One hour later, the absorbance at 450 nm was measured against a reagent blank. A standard curve was prepared with Trolox in 75% methanol–water containing 1% formic acid, and the results were expressed as mg of TE per mL sample.

### 4.7. Liquid Chromatography Tandem-Mass Spectrometry (LC-MS/MS) Analysis of Individual Phenolic Compounds

LC–MS/MS analysis was performed according to previously reported methods [50,53], with minor modifications. Polyphenolic compounds in propolis samples were analyzed using a Waters UPLC–MS/MS system (Milford, MA, USA) equipped with an Acquity BEH C18 column (1.7 µm, 50 × 2.1 mm). Prior to analysis, samples were injected at 10 µL. Chromatographic separation was achieved at a flow rate of 0.45 mL min^−1^ using a binary mobile phase consisting of 0.2% formic acid in ultrapure water (mobile phase A) and 0.1% formic acid in acetonitrile (mobile phase B). Gradient elution was applied, starting at 10% B, increasing to 50% B within 8 min, held for 2 min, and then returned to the initial conditions, resulting in a total run time of 12 min. The column and autosampler temperatures were maintained at 60 °C and 4 °C, respectively. Mass spectrometric detection was performed by electrospray ionization (ESI) in both positive and negative ion modes, using multiple reaction monitoring (MRM). In the negative ion mode, the capillary voltage was set to 3.0 kV, the desolvation temperature was set to 400 °C, the collision energy was 30 V, and the desolvation gas flow rate was 650 L h^−1^. Data acquisition was carried out using compound-specific *m*/*z* transitions. Instrumental parameters, including cone voltage and collision energy, were optimized individually for each analyte, and the corresponding MRM transitions are reported in Table A3.

### 4.8. Statistical Analysis

The effects of sample treatment on total phenolic and antioxidant properties were assessed scientifically using spectrophotometric data (TPC, TFC, and CUPRAC). The distributions of TPC, TFC, and CUPRAC departed from normality (*p* < 0.05 in most groups), according to the Shapiro–Wilk test, which evaluated the assumption of normality.

After comparing mean differences using one-way analysis of variance (ANOVA), specific pairwise differences (*p* < 0.05) were identified using Tukey’s HSD post hoc test. Additionally, correlations between phenolic/flavonoid contents (TPC and TFC) and antioxidant capacity (CUPRAC) were investigated using Pearson’s correlation analysis.

Boxplots were created to show the distribution of TPC, TFC, and CUPRAC by sample type, along with variation and possible outliers. Country-based visualization of spectrophotometric parameters was performed using a heatmap approach. Python (v3.10) with the pandas (v2.2), NumPy (v1.26), SciPy (v1.12), statsmodels (v0.14), and Matplotlib (v3.8) libraries was used to create all statistical analyses and graphical outputs.

After log-transforming and Pareto-scaling the raw LC-MS data variables, unsupervised Principal Component Analysis (PCA) was used to examine general sample clustering tendencies using the scikit-learn (v1.5) library in Python (v3.10) implemented in a Jupyter/Google Colab environment.

Given the heterogeneity of commercial products, unequal group sizes, and occasional deviations from normality, statistical analyses were applied in an exploratory manner to identify general trends rather than to derive definitive inferential conclusions.

## 5. Conclusions

This study provides a comprehensive evaluation of the phenolic content and antioxidant properties of commercial propolis products marketed in various liquid formats and sourced from multiple countries. Although the country of origin was initially considered a relevant variable, the present study’s findings demonstrate that formulation and product type exert a stronger influence on phenolic composition and antioxidant capacity in commercially available propolis products. Significant variation in phenolic content and antioxidant activity was observed by combining spectrophotometric tests with LC-MS-based targeted metabolomic profiling and multivariate analysis. The findings demonstrate how phenolic profiles and antioxidant activity are more significantly influenced by product type and formulation than by geographic origin alone. Physicochemical factors, including pH and moisture content, further contextualize the observed variability, while flavonoids and phenolic acids emerged as key contributors to sample differentiation and antioxidant activity. Overall, these findings highlight the need for improved labeling accuracy and greater standardization of propolis products to ensure quality and consumer transparency, as well as the importance of integrating targeted and untargeted analytical techniques for the characterization of complex natural products.

## Figures and Tables

**Table 1:** Minimum and maximum values of TPC, TFC, and CUPRAC for commercial propolis products by country, with the number of analyzed samples (*n*).

Region	Number of Samples (*n*)	TPC		TFC		CUPRAC	
		(mg GAE/mL Sample)		(mg CE/mL Sample)		(mg TE/mL Sample)	
		Min	Max	Min	Max	Min	Max
Belgium	3	1.7 ± 0.2	11.8 ± 2.0	0.2 ± 0.0	3.7 ± 0.6	3.1 ± 0.6	23.4 ± 2.9
Brazil	3	24.1 ± 4.9	34.2 ± 9.0	12.6 ± 1.9	19.5 ± 2.4	55.3 ± 10.7	83.9 ± 8.3
Croatia	2	0.9 ± 0.2	11.3 ± 1.5	0.3 ± 0.0	5.8 ± 1.0	1.7 ± 0.3	28.2 ± 4.7
Estonia	5	3.8 ± 0.6	10.9 ± 1.8	1.6 ± 0.4	5.5 ± 0.8	9.5 ± 1.2	24.2 ± 4.5
France	3	0.5 ± 0.1	2.3 ± 0.4	0.2 ± 0.1	0.8 ± 0.1	0.9 ± 0.2	3.6 ± 0.6
Germany	7	4.9 ± 0.7	18.8 ± 3.0	0.7 ± 0.1	6.2 ± 0.8	11.6 ± 1.7	54.8 ± 6.7
Italy	5	1.0 ± 0.1	2.7 ± 0.5	0.3 ± 0.0	1.0 ± 0.2	0.1 ± 0.0	5.0 ± 0.6
Japan	2	3.2 ± 0.4	5.8 ± 0.8	6.1 ± 1.8	10.5 ± 1.5	19.1 ± 2.2	29.1 ± 3.0
Korea	18	0.1 ± 0.0	107.5 ± 15.1	0.1 ± 0.0	40.9 ± 5.0	1.9 ± 0.4	351.4 ± 50.8
Romania	8	0.2 ± 0.1	17.3 ± 2.6	0.8 ± 0.1	11.4 ± 1.1	0.5 ± 0.1	48.2 ± 6.0
San Marino	2	29.3 ± 2.8	42.1 ± 5.3	4.9 ± 0.8	19.1 ± 1.9	50.4 ± 5.9	104.0 ± 10.4
Slovenia	4	1.6 ± 0.3	39.0 ± 4.2	0.8 ± 0.2	35.4 ± 5.5	3.4 ± 0.9	104.2 ± 20.2
Spain	2	3.9 ± 0.8	10.4 ± 1.8	4.2 ± 0.5	11.1 ± 1.1	44.4 ± 4.5	58.2 ± 10.0
UAE	6	0.1 ± 0.0	3.6 ± 0.4	0.1 ± 0.0	7.7 ± 1.0	0.1 ± 0.0	14.9 ± 2.4
USA	3	0.7 ± 0.1	101.2 ± 10.3	1.1 ± 0.2	174.5 ± 25.5	17.9 ± 1.9	492.3 ± 57.3
Latvia	1	2.7 ± 0.3		1.1 ± 0.2		6.3 ± 1.0	
New Zealand	1	93.5 ± 10.2		36.2 ± 5.2		204.0 ± 28.5	
Russia	1	13.1 ± 1.4		5.2 ± 1.0		29.2 ± 4.0	

Data represent average values ± standard deviation of three independent samples.

## Data Availability

The original contributions presented in this study are included in the article. Further inquiries can be directed to the corresponding author(s).

## Abbreviations

The following abbreviations are used in this manuscript:

ANOVA	Analysis of variance
CAPE	Caffeic acid phenethyl ester
CE	Catechin Equivalent
CUPRAC	Cupric Reducing Antioxidant Capacity
DPPH	2,2-diphenyl-1-picrylhydrazyl
GAE	Gallic Acid Equivalent
LC-MS/MS	Liquid chromatography–mass spectrometry
PCA	Principal Component Analysis
TE	Trolox Equivalent
TFC	Total Flavonoid Content
TPC	Total Phenolic Content
UAE	United Arab Emirates
UPLC–MS	Ultra-performance liquid chromatography-mass spectrometry
USA	United States of America

## Appendix A

**Table A1:** The information of samples regarding country of origin, solvent extraction, product type, pH, and dry matter.

Region	The Solvent of Extraction	Product Type	Sample No	pH	Dry Matter %
Belgium	Water	Spray	101	3.2 ± 0.3	42.8 ± 7.4
	Water + Glycerin	Spray	105	3.6 ± 0.5	48.3 ± 4.1
	Water	Syrup	115	3.3 ± 0.7	72.1 ± 11.9
Brazil	Ethanol	Drop	55	5.3 ± 0.4	12.2 ± 1.4
	Propylene glycol	Drop	49	5.5 ± 1.0	10.4 ± 1.9
	Water + Ethanol	Drop	63	4.5 ± 1.6	15.6 ± 1.6
Croatia	Water + Ethanol	Spray	34	5.1 ± 0.5	4.4 ± 0.6
	Water + Ethanol	Syrup	97	4.6 ± 0.8	39.8 ± 7.4
Estonia	Not Specified on the Label	Drop	2	-	0.1 ± 0.0
	Ethanol	Drop	28	5.2 ± 1.2	3.3 ± 0.2
	Not Specified on the Label	Spray	117	5.8 ± 0.7	90.6 ± 4.1
	Not Specified on the Label	Spray	118	5.4 ± 0.6	92.1 ± 10.6
	Water + Glycerin	Spray	65	4.4 ± 0.8	16.2 ± 1.7
France	Water	Other	91	3.8 ± 0.7	34.7 ± 1.1
	Water	Spray	25	3.7 ± 0.5	3.2 ± 0.7
	Water + Glycerin	Spray	108	3.5 ± 0.5	53.5 ± 5.7
Germany	Water	Drop	53	5.5 ± 0.5	10.8 ± 1.6
	Water	Drop	85	4.4 ± 1.0	32.4 ± 2.9
	Water + Ethanol	Drop	45	5.6 ± 0.6	10.0 ± 0.9
	Water + Ethanol	Drop	43	5.8 ± 1.1	8.6 ± 1.1
	Ethanol	Drop	35	4.5 ± 0.6	4.9 ± 0.5
	Not Specified on the Label	Other	70	3.7 ± 0.5	19.5 ± 2.8
	Water	Syrup	88	3.6 ± 0.4	33.6 ± 4.6
Italy	Water + Glycerin	Spray	103	3.9 ± 0.3	44.4 ± 5.4
	Water + Glycerin	Spray	37	7.7 ± 1.1	4.9 ± 0.8
	Water + Glycerin	Spray	95	4.4 ± 0.6	36.4 ± 7.9
	Water	Spray	31	4.7 ± 0.9	3.6 ± 0.8
	Water + Glycerin	Syrup	106	4.3 ± 1.0	49.1 ± 6.6
Japan	Not Specified on the Label	Drop	5	-	0.6 ± 0.1
	Water + Ethanol	Spray	94	4.6 ± 0.2	36.1 ± 7.1
Korea	Water	Drop	99	8.6 ± 0.5	41.1 ± 4.3
	Not Specified on the Label	Drop	120	6.2 ± 0.7	96.1 ± 10.0
	Propylene glycol	Drop	89	4.9 ± 0.8	33.7 ± 6.6
	Ethanol	Drop	75	5.5 ± 1.1	21.2 ± 3.5
	Propylene glycol	Drop	62	5.4 ± 0.8	14.8 ± 2.3
	Water + Ethanol	Drop	54	3.2 ± 0.6	11.0 ± 1.5
	Water	Other	1	-	0.1 ± 0.0
	Not Specified on the Label	Other	81	-	25.1 ± 3.6
	Water	Spray	42	9.3 ± 1.1	7.2 ± 1.0
	Water + Glycerin	Spray	102	7.3 ± 1.1	43.8 ± 0.9
	Water + Ethanol	Spray	82	5.8 ± 1.4	30.0 ± 5.6
	Water + Glycerin	Spray	112	4.1 ± 1.1	67.3 ± 3.3
	Glycerin + Ethanol + Propylene glycol	Spray	64	5.5 ± 0.7	15.8 ± 2.9
	Not Specified on the Label	Spray	72	3.6 ± 0.4	19.8 ± 1.1
	Glycerin + Ethanol + Propylene glycol	Spray	76	5.1 ± 0.2	21.3 ± 4.3
	Water + Glycerin	Spray	109	4.2 ± 0.3	55.4 ± 4.6
	Glycerin + Ethanol	Spray	100	6.0 ± 0.3	42.7 ± 6.5
	Water	Spray	79	4.1 ± 0.6	23.4 ± 2.9
Latvia	Water + Glycerin + Ethanol	Spray	52	5.2 ± 1.0	10.6 ± 1.9
New Zealand	Propylene glycol	Drop	69	4.9 ± 1.0	19.3 ± 3.6
Romania	Glycerin	Drop	116	4.7 ± 0.8	80.5 ± 8.6
	Water + Ethanol	Drop	41	4.2 ± 0.6	6.6 ± 0.7
	Water	Spray	83	3.6 ± 0.6	30.3 ± 6.2
	Water	Spray	86	3.5 ± 0.6	33.1 ± 3.5
	Water + Glycerin	Spray	110	4.6 ± 0.8	61.5 ± 8.9
	Water	Spray	39	3.5 ± 0.7	6.1 ± 1.1
	Water	Syrup	104	-	46.0 ± 1.6
	Glycerin	Syrup	107	3.8 ± 0.5	52.7 ± 3.0
Russia	Ethanol	Drop	61	5.4 ± 0.6	14.5 ± 3.1
San Marino	Water + Glycerin	Drop	68	3.2 ± 0.8	18.9 ± 3.9
	Water + Ethanol	Drop	77	4.6 ± 0.6	22.0 ± 3.2
Slovenia	Polietilen glikol	Drop	119	5.8 ± 0.4	94.8 ± 9.3
	Water + Glycerin + Ethanol	Drop	44	5.8 ± 0.2	8.9 ± 0.4
	Water + Glycerin	Spray	12	-	1.5 ± 0.3
	Water + Glycerin + Ethanol	Spray	96	5.5 ± 0.5	37.7 ± 3.0
Spain	Water + Glycerin	Drop	84	7.5 ± 0.3	31.8 ± 4.5
	Propylene glycol	Drop	36	3.6 ± 0.4	4.9 ± 0.7
UAE	Water	Other	113	-	68.9 ± 2.1
	Not Specified on the Label	Other	114	3.8 ± 1.0	71.9 ± 8.3
	Water	Spray	24	3.6 ± 0.7	3.1 ± 0.2
	Water	Spray	17	4.7 ± 0.7	2.2 ± 0.4
	Glycerin	Spray	22	5.0 ± 1.0	2.8 ± 0.6
	Water + Glycerin	Spray	90	4.7 ± 0.5	34.2 ± 4.2
USA	Ethanol	Drop	111	-	67.1 ± 7.8
	Not Specified on the Label	Drop	121	-	98.3 ± 11.3
	Not Specified on the Label	Spray	13	5.9 ± 1.2	1.5 ± 0.2

Data represent average values ± standard deviation of three independent samples. Samples having undetectable levels under the specified analytical conditions or missing formulation information on the product label are indicated by values reported as “-”.

**Table A2:** Quantitative LC-MS/MS results of propolis samples obtained from different commercial sources.

Group No	Sample No	Phenolic Acids					Flavones		Flavanones		Flavanols		
		p-Coumaric Acid	Gallic Acid	Caffeic Acid	CAPE *	Ferrulic Acid	Chrysin	Apigenin	Pinocembrin	Naringenin	Galangin	Cathechin	Quercetin
1	13	-	-	-	-	-	79.6 ± 15.2	1370.7 ± 257.5	157.6 ± 21.9	90.9 ± 20.3	2729.1 ± 505.3	-	57.9 ± 13.4
1	36	186.7 ± 37.3	-	320.8 ± 48.3	-	60.4 ± 11.9	-	-	-	56.2 ± 11.8	494.5 ± 56.5	-	105.7 ± 22.5
1	42	92.3 ± 18.0	-	-	250.4 ± 59.2	-	5090.8 ± 622.3	121.9 ± 28.7	2138.6 ± 205.3	-	-	-	-
1	49	485.6 ± 96.9	-	252.8 ± 51.8	121.5 ± 22.9	20.5 ± 3.8	6621.9 ± 1211.8	2204.3 ± 518.9	10,548.9 ± 2036.9	14.2 ± 3.2	5403.5 ± 647.0	-	90.8 ± 18.7
1	54	137.5 ± 26.2	-	49.5 ± 6.4	-	117.4 ± 22.5	-	1447.4 ± 326.8	5809.7 ± 826.1	152.0 ± 29.8	3183.0 ± 698.4	-	53.5 ± 12.7
1	55	675.9 ± 141.0	-	160.9 ± 38.1	-	41.6 ± 8.4	-	14.6 ± 3.3	225.8 ± 33.5	23.6 ± 4.4	-	-	126.0 ± 23.7
1	62	-	-	-	133.9 ± 26.2	-	6805.2 ± 1152.5	4520.6 ± 924.1	-	154.8 ± 32.3	8238.0 ± 814.3	-	62.7 ± 13.1
1	63	1449.7 ± 326.8	-	304.2 ± 66.8	-	66.4 ± 12.5	389.9 ± 82.9	992.1 ± 189.5	262.7 ± 55.7	15.0 ± 2.8	1876.5 ± 268.0	-	181.0 ± 34.3
1	64	-	-	168.2 ± 25.5	53.0 ± 12.3	-	1976.9 ± 381.8	757.2 ± 150.3	12.9 ± 2.7	21.2 ± 4.1	-	-	31.0 ± 5.7
1	68	12.2 ± 2.3	33.0 ± 4.6	-	76.4 ± 14.1	-	189.2 ± 27.2	273.7 ± 56.9	14.3 ± 2.1	47.5 ± 8.6	-	16.4 ± 3.3	18.5 ± 3.4
1	69	303.6 ± 43.5	-	1322.6 ± 255.1	170.0 ± 36.6	130.9 ± 29.2	4238.8 ± 704.6	529.2 ± 109.2	2162.1 ± 295.9	-	3332.9 ± 613.8	-	293.1 ± 56.6
1	70	-	-	-	-	-	58.8 ± 13.3	-	77.1 ± 15.6	-	-	-	-
1	75	-	-	-	14.0 ± 2.6	130.7 ± 24.8	19.3 ± 3.4	19.5 ± 4.3	-	50.0 ± 9.9	32.5 ± 5.4	-	21.8 ± 4.2
1	76	22.4 ± 4.9	-	12.7 ± 1.6	-	-	-	691.3 ± 152.2	4134.4 ± 399.9	105.2 ± 23.0	2408.6 ± 485.9	-	1365.2 ± 271.6
1	77	73.8 ± 17.6	418.7 ± 72.0	52.2 ± 5.7	53.6 ± 11.0	28.4 ± 6.2	617.8 ± 88.2	30.7 ± 6.2	830.9 ± 140.6	109.6 ± 18.4	-	314.3 ± 66.1	88.7 ± 16.7
1	82	-	-	-	94.8 ± 21.1	-	5627.9 ± 748.4	49.6 ± 10.7	187.3 ± 48.9	124.1 ± 27.1	-	-	143.0 ± 33.2
1	89	13.2 ± 3.0	-	-	-	-	-	-	2806.4 ± 268.6	179.6 ± 33.1	-	-	216.3 ± 45.0
1	99	237.3 ± 31.2	-	1451.5 ± 206.4	-	27.2 ± 5.5	-	1982.6 ± 439.0	59.5 ± 13.5	-	3643.4 ± 519.4	-	40.4 ± 7.6
1	100	-	-	-	16.0 ± 3.3	-	268.8 ± 40.8	1343.6 ± 251.0	72.0 ± 14.0	59.7 ± 11.6	4226.2 ± 808.0	-	24.8 ± 5.0
1	111	101.6 ± 20.8	-	56.8 ± 11.5	235.4 ± 53.2	32.5 ± 6.8	2417.7 ± 461.9	-	1783.5 ± 363.6	287.0 ± 53.1	-	-	241.3 ± 53.6
1	119	92.6 ± 22.0	-	44.6 ± 8.3	30.0 ± 5.6	87.1 ± 19.9	-	1370.1 ± 321.2	-	143.3 ± 29.5	79.2 ± 13.0	-	108.6 ± 20.1
1	120	-	-	-	-	-	-	-	-	84.2 ± 18.5	-	-	44.1 ± 8.8
2	1	-	-	-	-	-	-	-	-	-	-	-	-
2	2	-	-	-	-	-	-	-	-	-	-	-	-
2	5	-	87.8 ± 8.6	-	34.5 ± 4.6	-	32.6 ± 4.2	1082.9 ± 174.2	-	-	-	-	-
2	17	39.1 ± 7.1	-	-	-	-	137.2 ± 24.1	-	133.1 ± 17.1	-	-	-	-
2	22	-	-	-	-	-	-	-	-	-	-	-	-
2	24	-	-	-	11.3 ± 1.9	-	746.7 ± 39.5	-	-	-	-	-	-
2	25	-	-	-	-	-	-	-	-	-	-	-	-
2	28	217.7 ± 11.9	-	75.8 ± 13.3	-	204.9 ± 10.2	-	-	1852.0 ± 453.3	33.0 ± 6.0	-	-	17.6 ± 4.8
2	31	-	-	-	-	-	-	-	-	-	-	-	-
2	34	201.6 ± 4.5	12.1 ± 1.9	929.3 ± 10.4	448.3 ± 14.1	127.9 ± 8.6	7307.5 ± 555.0	767.0 ± 76.4	153.4 ± 5.4	-	1770.5 ± 431.2	59.0 ± 3.2	43.6 ± 8.1
2	35	498.9 ± 12.8	-	127.5 ± 13.0	-	421.4 ± 85.6	-	1410.4 ± 339.7	1994.7 ± 389.4	24.7 ± 6.6	-	-	-
2	37	-	-	-	-	-	-	-	-	-	-	-	-
2	39	-	-	-	-	-	-	-	-	-	-	-	-
2	41	202.9 ± 18.5	-	135.7 ± 17.4	452.0 ± 47.5	84.1 ± 12.3	-	-	2705.4 ± 669.5	38.2 ± 8.9	17.1 ± 2.6	-	32.1 ± 6.0
2	43	21.1 ± 3.9	-	45.4 ± 12.4	16.8 ± 2.3	-	-	-	8634.2 ± 1317.8	94.0 ± 10.5	-	-	48.5 ± 10.9
2	44	106.9 ± 13.6	-	75.5 ± 12.4	297.9 ± 15.5	49.0 ± 5.7	4632.3 ± 830.3	1943.3 ± 430.7	2386.7 ± 287.1	122.9 ± 14.5	4266.5 ± 573.8	-	94.4 ± 9.9
2	45	53.0 ± 10.9	-	71.3 ± 10.3	-	24.6 ± 5.6	1757.7 ± 391.8	41.4 ± 4.9	926.3 ± 45.4	88.6 ± 15.4	144.8 ± 6.2	-	47.3 ± 8.3
2	52	279.8 ± 52.5	-	27.5 ± 3.8	342.0 ± 7.5	238.3 ± 34.6	-	-	-	-	-	-	-
2	53	160.3 ± 7.0	-	12.3 ± 1.2	-	-	3487.2 ± 308.0	848.7 ± 133.4	22.2 ± 6.1	-	1815.4 ± 321.3	-	-
2	61	97.7 ± 6.8	-	34.5 ± 7.8	-	23.3 ± 5.1	-	308.8 ± 15.3	123.6 ± 9.4	81.0 ± 8.4	483.7 ± 43.2	-	39.9 ± 5.9
2	65	406.3 ± 24.3	-	60.4 ± 13.8	21.6 ± 6.1	502.2 ± 36.7	-	554.8 ± 56.3	37.7 ± 8.4	35.4 ± 6.0	388.7 ± 27.2	-	-
2	72	-	-	-	16.2 ± 3.3	-	17.2 ± 1.8	-	13.8 ± 0.8	-	11.2 ± 2.3	-	-
2	79	-	-	-	24.4 ± 5.3	-	25.3 ± 4.9	22.2 ± 4.3	18.7 ± 4.5	-	24.8 ± 4.6	-	-
2	81	-	-	-	-	-	-	-	-	-	-	-	-
2	83	520.9 ± 19.5	-	467.7 ± 56.3	-	481.6 ± 41.2	-	-	-	35.3 ± 6.3	-	-	39.9 ± 7.6
2	84	-	-	-	-	-	748.9 ± 97.6	-	81.4 ± 11.3	-	-	-	-
2	85	-	-	-	-	-	-	90.6 ± 16.2	14.2 ± 3.6	-	69.2 ± 14.3	-	-
2	86	265.5 ± 44.5	-	344.2 ± 95.4	-	210.4 ± 13.1	-	104.4 ± 8.8	19.3 ± 3.4	33.6 ± 4.9	-	-	26.3 ± 4.7
2	88	-	-	-	-	-	-	-	-	-	-	-	-
2	90	-	-	-	40.3 ± 10.1	-	34.1 ± 2.4	94.3 ± 11.6	31.3 ± 6.2	-	126.4 ± 10.6	-	-
2	91	-	-	-	-	-	-	-	-	-	-	-	-
2	94	375.4 ± 10.9	-	99.6 ± 7.2	488.3 ± 33.8	14.2 ± 3.3	3470.3 ± 487.9	4461.1 ± 776.6	33.1 ± 8.8	-	9872.6 ± 213.3	-	34.9 ± 6.2
2	95	-	-	-	-	-	-	-	-	12.4 ± 2.7	-	-	-
2	96	-	-	-	-	-	-	-	-	-	-	-	-
2	97	-	-	-	-	-	-	-	-	-	-	-	-
2	101	-	21.3 ± 4.4	-	-	-	43.3 ± 7.2	-	-	17.2 ± 4.4	-	-	-
2	102	-	-	-	170.0 ± 7.6	-	4381.3 ± 347.4	1332.9 ± 197.8	3422.6 ± 536.4	-	3.419 ± 159	-	-
2	103	113.5 ± 18.6	-	113.9 ± 11.4	-	37.6 ± 6.8	-	-	385.0 ± 25.0	42.8 ± 6.5	-	-	27.5 ± 4.5
2	104	-	-	11.2 ± 1.3	491.1 ± 76.1	-	3202.4 ± 504.1	22.3 ± 3.9	-	-	-	-	-
2	105	46.4 ± 4.4	-	13.0 ± 2.8	-	10.9 ± 2.5	28.0 ± 4.7	268.1 ± 40.5	-	46.1 ± 4.9	193.1 ± 28.3	-	22.1 ± 5.2
2	106	-	-	-	-	-	-	-	710.6 ± 100.4	-	-	-	-
2	107	-	-	61.0 ± 13.0	18.5 ± 4.3	-	391.6 ± 50.9	1245.9 ± 264.6	1129.3 ± 262.1	-	2849.0 ± 328.8	-	-
2	108	51.4 ± 8.9	-	20.9 ± 4.6	38.5 ± 9.0	11.7 ± 2.2	522.2 ± 8.9	-	527.7 ± 73.1	45.2 ± 7.1	-	-	24.1 ± 4.7
2	109	-	-	-	183.6 ± 23.3	-	5110.5 ± 415.3	71.2 ± 15.3	2245.6 ± 406.4	-	-	-	-
2	110	56.5 ± 13.1	-	44.8 ± 10.1	29.2 ± 5.6	17.0 ± 3.0	-	-	-	63.0 ± 9.0	-	-	-
2	112	34.4 ± 7.5	-	41.6 ± 7.8	225.6 ± 29.0	-	10,985.1 ± 1124.9	1950.8 ± 199.9	2502.2 ± 609.7	29.3 ± 2.2	-	-	-
2	113	-	-	-	-	-	-	-	-	-	-	-	-
2	114	-	-	-	-	-	-	26.6 ± 6.6	-	10.0 ± 2.1	22.6 ± 4.2	-	-
2	115	-	-	-	-	-	102.4 ± 17.2	-	20.4 ± 4.7	-	-	-	-
2	116	461.3 ± 41.3	-	90.4 ± 5.9	107.8 ± 10.4	-	7496.5 ± 754.9	1662.5 ± 546.5	2959.0 ± 339.3	24.3 ± 4.0	3857.3 ± 217.8	-	59.8 ± 12.6
2	117	205.8 ± 30.9	-	12.3 ± 2.2	-	217.2 ± 40.5	-	-	744.4 ± 82.6	13.8 ± 2.5	-	-	-
2	118	187.6 ± 19.2	-	14.0 ± 1.5	462.8 ± 46.9	224.2 ± 18.4	-	627.2 ± 92.4	34.9 ± 9.5	16.9 ± 1.9	409.4 ± 16.5	-	-
2	121	-	-	-	-	-	-	-	-	-	-	-	-

Quantitative LC-MS/MS results for 23 selected propolis samples that exhibited 50 mg Trolox per mL by CUPRAC assay were classified as group 1, and the rest of the samples were classified as group 2. Data represent average values ± standard deviation of three independent samples. Results were given as mg standard per mL sample. * CAPE: Caffeic acid phenethyl ester.

**Table A3:** Multiple reaction monitoring (MRM) parameters for the targeted polyphenolic compounds analyzed by UPLC–MS/MS.

Compound	Ionization Mode	Precursor Ion (*m*/*z*)	Product Ion (*m*/*z*)	Cone Voltage (V)	Collision Energy (eV)	Dwell Time (s)
Chrysin	ESI(+)	255.03	66.51	64	34	0.011
		255.03	153.00	64	30	0.011
Pinocembrin	ESI(+)	257.00	153.00	42	22	0.011
Galangin	ESI(+)	271.03	104.94	62	30	0.011
		271.03	153.00	62	34	0.011
p-Coumaric acid	ESI(−)	162.84	92.85	32	24	0.011
		162.84	118.86	32	16	0.011
Gallic acid	ESI(−)	169.00	53.20	35	20	0.013
		169.00	79.30	35	20	0.013
		169.00	97.30	35	20	0.013
		169.00	125.20	35	15	0.013
Caffeic acid	ESI(−)	179.30	107.10	30	25	0.011
		179.30	135.20	30	15	0.011
		180.84	108.00	40	22	0.011
Ferulic acid	ESI(−)	192.90	133.90	36	16	0.011
		192.90	177.90	36	12	0.011
Apigenin	ESI(−)	269.07	117.00	54	35	0.011
		269.07	151.00	54	35	0.011
Naringenin	ESI(−)	271.05	118.80	54	24	0.011
		271.05	150.00	54	20	0.011
CAPE	ESI(−)	283.10	135.00	54	24	0.011
		283.10	179.10	54	16	0.011
Catechin	ESI(−)	289.06	108.96	40	26	0.011
		289.06	245.09	40	14	0.011
Quercetin	ESI(−)	301.03	107.01	46	30	0.036
		301.03	151.03	46	22	0.036
		301.03	178.99	46	18	0.036
